# HNF4α and CDX2 Regulate Intestinal *YAP1* Promoter Activity

**DOI:** 10.3390/ijms20122981

**Published:** 2019-06-18

**Authors:** Sylvester Larsen, Johanne Davidsen, Katja Dahlgaard, Ole B. Pedersen, Jesper T. Troelsen

**Affiliations:** 1 Department of Science and Environment, Roskilde University, Universitetsvej 1, 4000 Roskilde, Denmark; sylvesterlarsen@outlook.com (S.L.); johadav@ruc.dk (J.D.); katja.d@hlgaard.dk (K.D.); 2 Department of Clinical Immunology, Næstved Hospital, Ringstedgade 77B, 4700 Næstved, Denmark; olbp@regionsjaelland.dk; 3 Department of Surgery, Center for Surgical Science, Enhanced Perioperative Oncology (EPEONC) Consortium, Zealand University Hospital, Lykkebækvej 1, 4600 Køge, Denmark

**Keywords:** Hippo pathway, intestinal epithelium, yes-associated protein 1 (YAP1), transcriptional regulation, gene regulation, transcription factor, cellular regulation

## Abstract

The Hippo pathway is important for tissue homeostasis, regulation of organ size and growth in most tissues. The co-transcription factor yes-associated protein 1 (YAP1) serves as a main downstream effector of the Hippo pathway and its dysregulation increases cancer development and blocks colonic tissue repair. Nevertheless, little is known about the transcriptional regulation of *YAP1* in intestinal cells. The aim of this study to identify gene control regions in the *YAP1* gene and transcription factors important for intestinal expression. Bioinformatic analysis of caudal type homeobox 2 (CDX2) and hepatocyte nuclear factor 4 alpha (HNF4α) chromatin immunoprecipitated DNA from differentiated Caco-2 cells revealed potential intragenic enhancers in the *YAP1* gene. Transfection of luciferase-expressing *YAP1* promoter-reporter constructs containing the potential enhancer regions validated one potent enhancer of the *YAP1* promoter activity in Caco-2 and T84 cells. Two potential CDX2 and one HNF4α binding sites were identified in the enhancer by in silico transcription factor binding site analysis and protein-DNA binding was confirmed in vitro using electrophoretic mobility shift assay. It was found by chromatin immunoprecipitation experiments that CDX2 and HNF4α bind to the *YAP1* enhancer in Caco-2 cells. These results reveal a previously unknown enhancer of the *YAP1* promoter activity in the *YAP1* gene, with importance for high expression levels in intestinal epithelial cells. Additionally, CDX2 and HNF4α binding are important for the *YAP1* enhancer activity in intestinal epithelial cells.

## 1. Introduction

The Hippo pathway is a signaling pathway that is important for tissue homeostasis, regulation of organ size and tumorigenesis [1,2]. It acts as a switch between proliferation and differentiation [1,3]. Extra- and intracellular growth signals are relayed through the Hippo phosphorylation kinase cascade that inactivates the two downstream effectors Yes-associated protein 1 (YAP1) and WW domain containing transcription regulator 1 (WWTR1). YAP1 is a co-transcription factor that interacts with the TEA domain transcription factor (TEAD) family members to regulate the expression of many genes, especially those important for proliferation, adhesion, migration and the extracellular matrix organization [4,5]. YAP1 is expressed in all human tissues, with a relatively medium expression in the intestinal tissue [6].

The activity of YAP1 is affected by protein–protein interactions, phosphorylation, and re-localization of YAP1. However, little is known about the regulation of *YAP1* on the transcriptional level. SRY-box transcription factor 2 (SOX2) has been shown to be a direct transcriptional regulator of *YAP1* in osteoprogenitor cells, where SOX2 activates transcription of *YAP1* without being essential for *YAP1* expression [7]. A study in hepatocellular carcinoma cells showed that knockdown of the heparan sulfate proteoglycan Glypican-3 decreased *YAP1* expression [8]. Both transcriptional regulator ERG (ERG) and TEAD4 can interact with the *YAP1* promoter and increase H3K9/14Ac acetylation which leads to increased *YAP1* expression in prostate tumors [9]. Furthermore, methylation of the *YAP1* promoter in polycystic ovary syndrome (PCOS) was also shown to increase YAP1 protein levels [10].

As typical for key regulators of proliferation, YAP1 has been linked to cancer progression. Several studies show that dysregulation of YAP1 is important in the development of colorectal cancers [11,12,13]. Furthermore, nuclear localization and overexpression of YAP1 have been correlated with a poor prognosis in colorectal cancers and other cancer types [14,15]. Recently, YAP1 was shown to be necessary for colonic tissue repair and remodeling by mediating cell reprogramming [16].

Several signaling pathways interact within intestinal epithelial cells, adding layers of complexity to the fine-tuning of the final transcriptional regulation. The main pathways involved in the differentiation of intestinal stem cells to the various cell lineages of the gut epithelium and homeostasis maintenance is Wnt/β-catenin, Notch, Hedgehog, epidermal growth factor receptor (EGFR) and Bone morphogenetic protein/transforming growth factor beta (Bmp/TGFβ), reviewed in [17,18]. The Hippo pathway converges with several of these signaling pathways [2,19]. Well-defined networks of intestinal epithelium-specific transcription factors control differentiation and proliferation of the intestinal epithelium [17,20,21]. Two key regulators in these networks are Caudal Type Homeobox 2 (CDX2) and Hepatocyte Nuclear Factor 4 alpha (HNF4α).

CDX2 is intestinal specific in adult mammals and is essential in the development and maintenance of intestinal tissue [22,23]. Ablation of CDX2 in in vitro intestinal organoid models leads to the development of gastric-like tissue lacking intestine-specific enzymes [24]. Dysregulation of CDX2 has been linked to colorectal cancer development and affects the severity of disease [25,26]. Mice with decreased CDX2 expression develop malignant tumors in the colon when treated with the DNA mutagen azoxymethane [27]. In humans, CDX2 levels are generally lower for late-stage adenocarcinomas and tumors with low or moderate differentiation levels [28,29].

HNF4α is highly expressed in the intestines but is also found in the liver, stomach, pancreas, kidney, and testis [30]. HNF4α is essential for the formation of crypts in the colon and is important for the development of the intestinal epithelium [31]. Furthermore, it regulates the expression of several enzymes and regulators involved in metabolism in the adult gut epithelium [32,33]. HNF4α also plays a role in cancer development but it is not well understood. One study found that some HNF4α isoforms are lost in 40% of colorectal cancers, while another study found a general upregulation of HNF4α in their samples [34,35]. 

CDX2 and HNF4α regulate each other and also auto-regulate their own expression in the intestines [36,37,38,39]. Additionally, several other transcription factors are important in the intestinal transcription factor network regulating intestinal development, such as the GATA-binding proteins (GATA4/5/6), HNF1 homeobox proteins (HNF1A/B), and transcription factor 7 like 2 (TCF7L2) [17]. CDX2 interacts with the Wnt/β-catenin pathway and might be an active component of it [40].

Previous studies of the Hippo pathway have predominantly focused on the protein–protein interactions of key kinases, which constitutes the core components of the pathway, and the role of the main effector proteins YAP1 and WWTR1. Little is known about how the transcription of *YAP1* is controlled in intestinal tissue. This study describes a novel mechanism of transcriptional regulation of *YAP1* in intestinal cells, by the transcription factors CDX2 and HNF4α.

## 2. Results

### 2.1. The Chromatin Landscape of the YAP1 Gene

**Figure 1:**
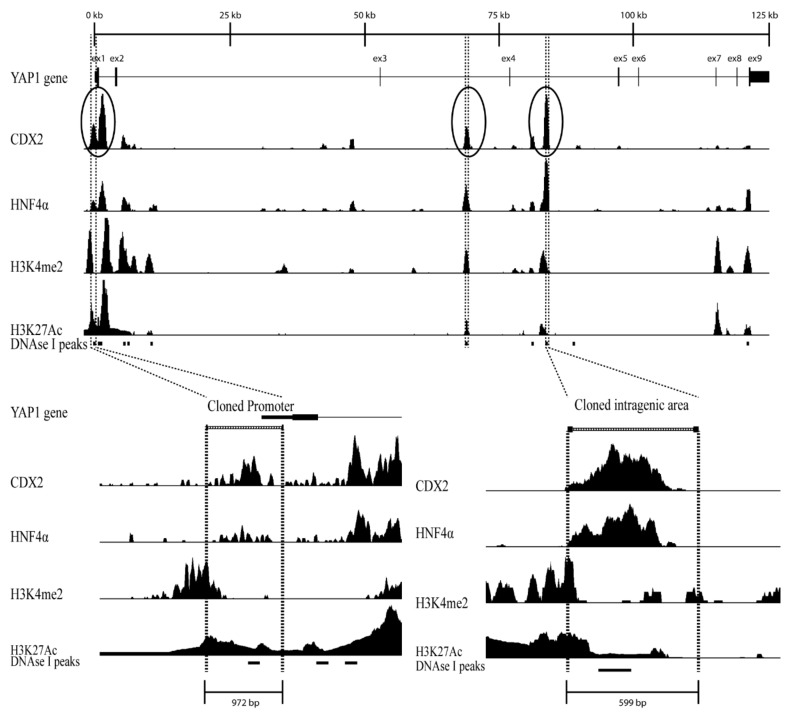
Putative regulatory regions within the *YAP1* gene; (**Top**) View of the *YAP1* gene region in the February 2009 GRCh37/hg19 genome assembly at http://genome.ucsc.edu. ChIP-seq data for CDX2, HNF4α, H3K4me2, and H3K27Ac, from confluent Caco-2 cells is shown as density graphs [39]. Circles and dashed lines indicate the promoter and intragenic regulatory regions in the *YAP1* gene; (**Bottom Left**) View of the promoter region of the *YAP1* gene spanning 972 bp (−0.7 kb to +0.3 kb relative to the transcription start site (TSS)); (**Bottom right**) View of the intragenic regulatory region in intron 4 of the *YAP1* gene spanning 599 bp (+82.2 kb to +82.8 kb relative to the TSS). The black bars in the DNase I track [42] from Caco-2 cells represent a significant increase in DNase sensitivity in these areas.

To identify regulatory regions in the *YAP1* gene chromatin immunoprecipitated followed by sequencing (ChIP-seq) data from differentiated Caco-2 cells were analyzed for the relative abundance of CDX2 and HNF4α. The ChIP-seq data analyzed were from Caco-2 cell precipitates with antibodies for the transcription factors CDX2 and HNF4α as well as for histone acetylation H3K27Ac and methylation H3K4me2 [41]. The *YAP1* gene was analyzed by aligning all data tracks and then identifying gene-regulatory regions by comparing the signal intensity from the ChIP-seq data for transcription factors and the DNase I track which is an indicator for transcriptionally active regions (Figure 1).

Three regions with clear CDX2 ChIP-seq peaks were chosen for further analysis, one region was in the *YAP1* promoter, one in intron 3 and another was in intron 4. The chosen *YAP1* promoter region spanned 972 bp located at -0.7 kb to +0.3 kb relative to the TSS (Figure 1). Clear ChIP-seq peaks were detected for both CDX2, HNF4α, and H3K27Ac in the promoter region, while the H3K4me2 peak was found directly adjacent to the region. 

The *YAP1* intragenic region in intron 4 spanned 599 bp located at +82.2 kb to +82.8 kb relative to the TSS (Figure 1). The intragenic region contained clear ChIP-seq signals for CDX2 and HNF4α, while both H3K4me2 and H3K27Ac peaks were found directly adjacent. 

The *YAP1* intragenic region in intron 3 spanned 632 bp located at +67.5 kb to +68.3 kb relative to the TSS (Figure 1). All three regions also contain a DNase I sensitivity signal which together with the ChIP-seq data indicate that these are regulatory active regions (Figure 1).

#### 2.1.1. *YAP1* Promoter Activity

To analyze the gene-regulatory activity of the *YAP1* intragenic region in intron 3 (+62 kb) and 4 (+82 kb) we used promoter-reporter assay analysis. We constructed luciferase reporter plasmids containing the *YAP1* promoter with or without the intragenic regions and transfected them into the intestinal cell lines Caco-2, T84, LS174T, DLD-1, SW480, and HT-29, as well as into non-intestinal HEK293 cells to test the *YAP1* promoter activity. The cells were transfected with *YAP1* reporter plasmids with or without the intragenic regions inserted, using an empty pGL4.10 vector as a negative control and *Lac*Z expression plasmid for transfection efficiency control. The region in intron 3 (+62 kb) did not show any enhancer activity (data not shown), thus we focused on characterizing the region from intron 4 in further in vitro analyses.

**Figure 2:**
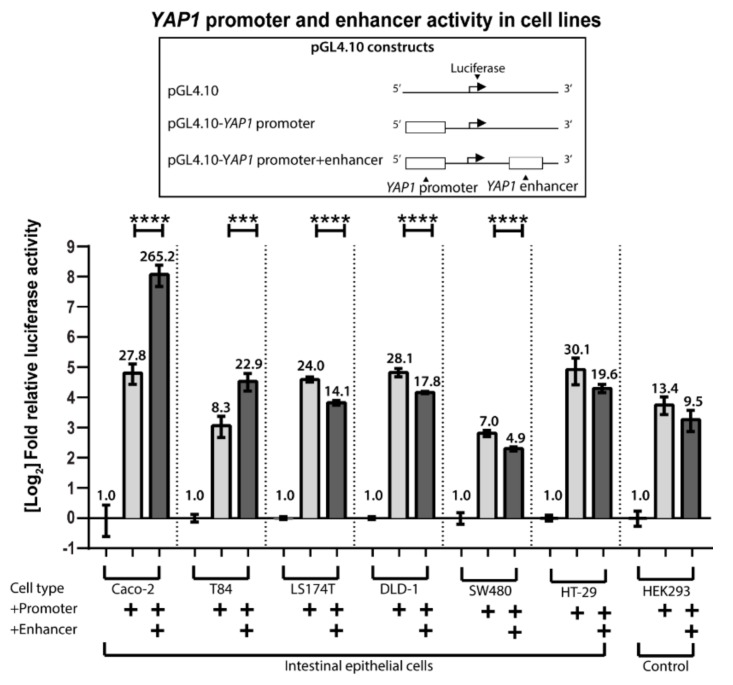
Relative luciferase/β-galactosidase activity from cell lysates of the intestinal cell lines Caco-2, T84, LS174T, DLD-1, SW480, and HT-29 transfected with pGL4.10 luciferase reporter constructs containing the *YAP1* promoter and +82 kb enhancer. HEK293 cells are used as a non-intestinal control. Vertical lines separate data from independent assays. Bars are mean values on a log2 scaled *y*-axis, with error bars showing SD, *n* = 4). + indicates that either the *YAP1* promotor or enhancer sequence was inserted in to the luciferase construct sequence. Statistical significance was obtained using one-way ANOVA with *p*-values under 0.001 (***) and 0.0001 (****)

pGL4.10 generated a very low background signal in all cell lines (Figure 2). Luciferase activity of the pGL4.10-*YAP1* promoter construct was 7 to 30-fold above background for all of the cell lines, revealing that the *YAP1* promoter is active in both intestinal and non-intestinal cells.

Transfections with the pGL4.10-*YAP1* promoter+enhancer (+82 kb intragenic region) construct in Caco-2 and T84 cells showed an increase in reporter gene activity compared to pGL4.10-*YAP1* promoter levels of 9.5-fold, and 2.7-fold, respectively. No increase in activity was detected in any of the other cell lines. Together, this data shows that the +82 kb intragenic region functions as an enhancer of *YAP1* in Caco-2 and T84 cells. Interestingly, these two cells lines are the only intestinal cell lines that share the ability to spontaneously differentiate upon confluency.

Analysis of ChIP-seq data from Caco-2 cells revealed that CDX2 and HNF4α are associated with regions in the *YAP1* gene and thus might be potential regulators of the expression of the *YAP1* gene. Locating specific binding sites for CDX2 and HNF4α in the *YAP1* promoter or +82 kb enhancer is necessary to be able to determine if they exert their regulation by specifically binding to the DNA. 

The JASPER algorithm predicted two CDX2 sites, and two HNF4α sites in the promoter region while three CDX2 and two HNF4α sites were found in the +82 kb enhancer by using the transcription factor binding site matrices MA04665.1 for CDX2 and MA0114.2 for HNF4α (Table S1).

Due to their high scores in the in silico analysis, three sites in the *YAP1* enhancer were selected for further analysis. These were two CDX2 sites (“CDX2-S1” and “CDX2-S2”) and one HNF4α site (“HNF4α”).

**Figure 3:**
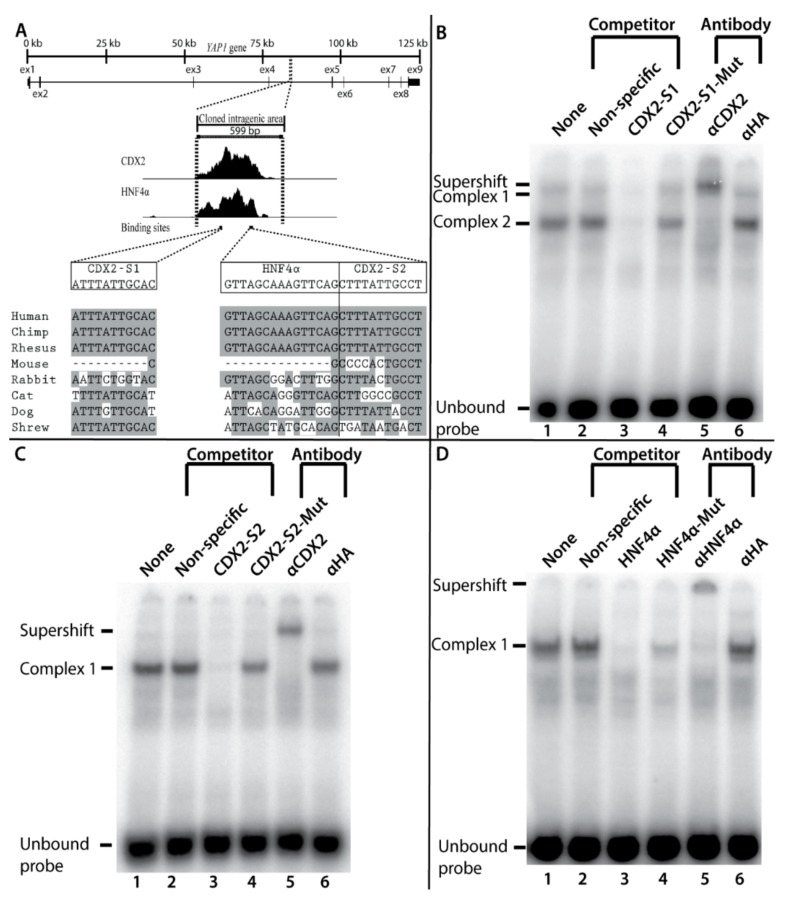
Electrophoretic mobility shift assay and conservation analysis of two CDX2 binding sites and one HNF4α binding site in the *YAP1* +82 kb enhancer named CDX2-S1 and CDX2-S2 and HNF4α. (**A**) Alignment of eight vertebrate sequences to identify evolutionary conservation at three detected transcription factor binding sites in the *YAP1* +82 kb enhancer. The dotted lines indicate the borders of the zoomed in-areas and the grey color indicates fully conserved bases that are identical to the human bases; (**B,C,D**) All samples contain: 1 μL nuclear extract from differentiated Caco-2, 2.5 fmol radioactive labeled probe (unbound probe is seen in the bottom of the image), and 0.5 μg poly(dI-dC) as a competitor for nonspecific DNA binding proteins. (**B**) In addition, lane 2 contains a non-specific DNA-oligo competitor; lane 3 contains non-labeled CDX2-S1 competitor oligo; lane 4 contains mutated non-labeled competitor oligo CDX2-S1-Mut; lane 5 contains CDX2 specific antibody (αCDX2); lane 6 contains anti-hemagglutinin (α HA) control antibody; (**C**) Same as in B, but with CDX2-S2 oligo and CDX2-S2-Mut oligo; (**D**) Same as in C, but with HNF4α oligo, HNF4α-Mut oligo, and anti-HNF4α antibody (αHNF4α).

To determine the potential importance of the three putative binding sites we aligned genomic DNA sequences from eight vertebrate species and compared the evolutionary conservation of the binding sites on the basis of the transcription factor binding site matrices for CDX2 and HNF4α (Figure 3A). The analysis showed conservation among the analyzed vertebrates except for in mice, in which the binding sites are not preserved. 

We used electrophoretic mobility shift assay (EMSA) to determine whether the two CDX2 and the one HNF4α in silico predicted transcription factor binding sites were functional and could bind endogenous proteins in Caco-2 cells. Oligonucleotides for each of the three binding sites were designed and 32P-radiolabeled. Unlabeled oligonucleotides with mutated binding sequences and a non-specific competitor were used as competitors (Table S3).

Clear specific complexes were formed on all three EMSAs when radiolabeled oligonucleotides were combined with the Caco-2 nuclear extract. Unlabeled oligonucleotides were able to compete for the binding of the radiolabeled oligonucleotides for all three sites, while the non-specific competitor was not which indicates that the protein-oligonucleotide complexes are specific.

Two protein-DNA complexes were formed between the oligonucleotide containing the CDX2-S1 site (Figure 3B), and proteins in the Caco-2 extract, while only one complex was formed using the CDX2-S2, or HNF4α radiolabeled oligonucleotides (Figure 3C) and (Figure 3D), respectively. Oligonucleotides with mutated binding sites were not able to compete for binding with the radiolabeled probe. However, the mutation in the HNF4α site did not completely destroy the binding ability for the mutated oligonucleotide as it slightly reduced binding to the probe (Figure 3D, lane 4).

When antibodies to CDX2, HNF4α or a negative control hemagglutinin (HA) were added, only CDX2 and HNF4α produced supershifts, demonstrating that they are part of the protein-radiolabeled oligonucleotide complexes. In conclusion, the EMSA revealed two CDX2 and one HNF4α binding sites in the *YAP1* +82 kb enhancer.

**Figure 4:**
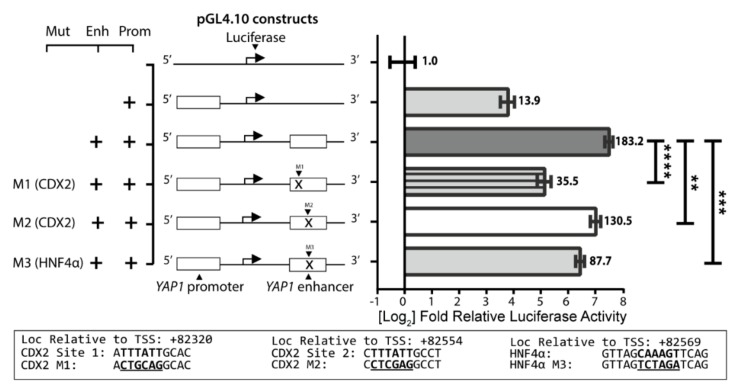
Relative luciferase/β-galactosidase activity from cell lysates of Caco-2 cells transfected with pGL4.10 luciferase reporter constructs containing the *YAP1* promoter and +82 kb enhancer with or without mutated CDX2 and HNF4α binding sites. The sequence of the three mutated binding sites CDX2 site 1 (M1), CDX2 site 2 (M2), and HNF4α (M3) is depicted below on the figure. Relative luciferase activity was normalized to the empty pGL4.10 vector. Bars are mean values on a log2 scaled *y*-axis with SD error bars. + indicates the presence of a YAP1 promotor or enhancer sequence in the luciferase reporter sequence. The bold bases at the bottom indicates the core sequence of the transcription factor binding site and mutated core sequences are underlined. Statistical significance was obtained using two-tailed unpaired student’s *T*-test with *p*-values under 0.01 (**), 0.001 (***), and 0.0001 (****), *n* = 4.

We wanted to investigate the effect of CDX2 and HNF4α on the *YAP1* +82 kb enhancer activity in Caco-2 cells. This was done by mutating the two CDX2 and one HNF4α binding sites that were confirmed to be functional in the EMSA analysis, in the pGL4.10-*YAP1* promoter+enhancer constructs by site-directed mutagenesis (Figure 4). Three constructs were created by mutation of three binding sites. CDX2-S1 (M1), CDX2-S2 (M2) and HNF4α (M3) were constructed and assayed for luciferase activity. The CDX2-M1 construct decreased the +82 kb enhancer effect more than 5-fold, *p* < 0.0001, while CDX2-M2 only decreased activity by 1.4-fold, *p* < 0.01. Removing the HNF4α site (M3) caused a 2-fold decrease in activity. This shows that the CDX2-S1 site is important for the +82 kb enhancer activity in Caco-2 cells and that both CDX2-S2 and the HNF4α site also contribute to the activity.

**Figure 5:**
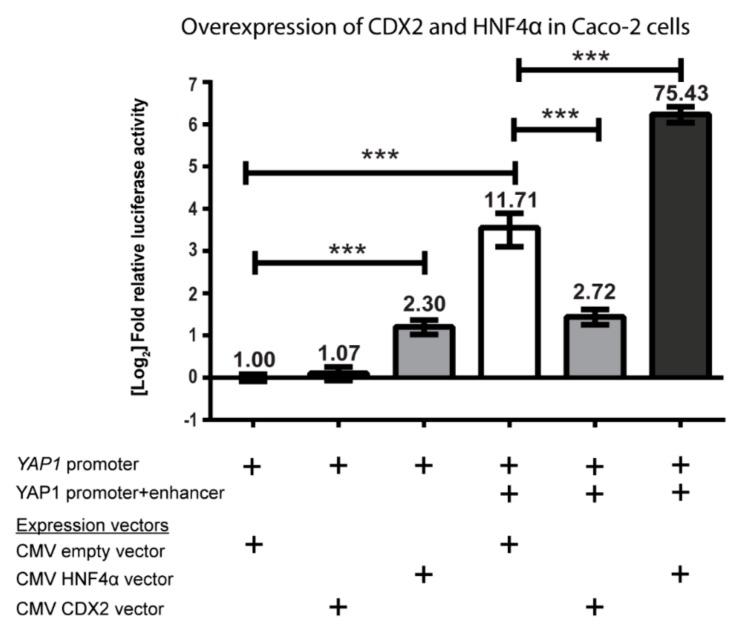
Relative luciferase/β-galactosidase activity from cell lysates of Caco-2 cells transfected with pGL4.10 luciferase reporter constructs containing the *YAP1* promoter and/or +82 kb enhancer. Cytomegalovirus (CMV)-HNF4α, CMV-CDX2 expression vector or an empty CMV vector were co-transfected with the pGL4.10 plasmids for the overexpression of HNF4α and CDX2. The relative luciferase/β-galactosidase activity was normalized to the pGL4.10-*YAP1* promoter construct. Bars are mean values plotted on a log2 scaled y-axis, with error bars showing 95% CI. + indicates the presence of a YAP1 promotor or enhancer sequence in the luciferase reporter sequence, or the addition of a CMV-based expression vector to the transfection. Data are statistically significant using two-tailed unpaired student’s *T*-test with *p*-values under 0.001 (***), *n* = 4.

Next, we performed an overexpression study, where the pGL4.10-*YAP1* promoter plasmid with or without the +82 kb enhancer was co-transfected with CDX2 and HNF4a expression plasmids to elucidate their effect on the transcriptional regulation of *YAP1* in Caco-2 cells. The relative luciferase activity was normalized to the pGL4.10-*YAP1* promoter plasmid. The activity of the pGL4.10-*YAP1* promoter plasmid was not affected by overexpression of CDX2 but was increased 2-fold by HNF4α overexpression (Figure 5). 

Overexpression of HNF4α with the pGL4.10-*YAP1* promoter+enhancer plasmid increased the activity of the already powerful +82 kb enhancer more than 6-fold, yielding more than a 75-fold difference from the pGL4.10-*YAP1* promoter plasmid alone. It was clear that HNF4α acted as a strong activator of *YAP1* expression via the *YAP1* +82 kb enhancer. Interestingly, overexpression of CDX2 did not increase the activity of the pGL4.10-*YAP1* promoter+enhancer plasmid but instead displayed an inhibitory role towards the activity. This result does not correlate with the decrease in activity that was seen when the CDX2 binding sites were mutated (Figure 4). The lack of effect when overexpressing CDX2 and analyzing enhancer activity in Caco-2 cells has previously been observed with other CDX2 regulated genes [43].

#### 2.1.2. CDX2 and HNF4α Binding Sites in the *YAP1* Intragenic Enhancer

**Figure 6:**
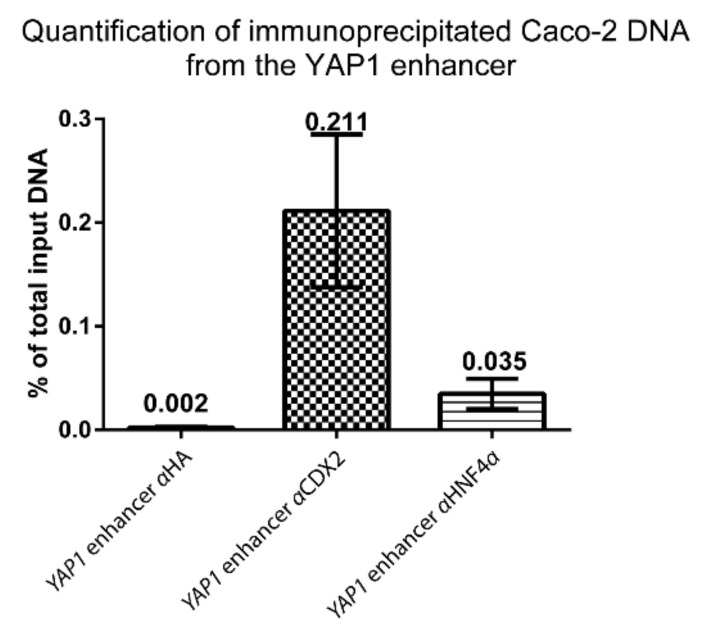
PCR quantification of Caco-2 DNA immunoprecipitated with either anti-CDX2 (αCDX2) or anti-HNF4α (αHNF4α) or control anti-hemagglutinin (αHA) antibodies. The amount of purified Caco-2 ChIP DNA was compared to the amount of input DNA (non-immunoprecipitated) in the *YAP1* +82 kb enhancer, using specific primers (Table S2-B). Quantification was performed by ChIP-qPCR and bars represent mean values with range, *n* = 2.

We performed quantitative PCR on ChIP DNA from Caco-2 cells to determine the relative abundance of CDX2 and HNF4α bound to the *YAP1* +82 kb enhancer (Figure 6). The relative abundance of immunoprecipitated DNA was compared to negative control DNA precipitated with Hemagglutinin (HA) antibody. Primers were designed to span a small region in the *YAP1* +82 kb enhancer with a clear ChIP-seq peak for CDX2 and HNF4α (Table S2-B). The relative abundance of *YAP1* +82 kb enhancer CDX2 immunoprecipitated DNA was 0.211% of total input DNA, which was a more than a 100-fold increase over the HA control ChIP level (0.002%). For HNF4α the relative abundance was 0.035% of total input DNA, which was a ~17-fold increase over the HA control ChIP level. Both CDX2 and HNF4α binding were substantially increased compared to the negative control which indicates that both transcription factors bind to the *YAP1* +82 kb enhancer in Caco-2 cells.

#### 2.1.3. CDX2 Activation of YAP1 Protein Expression

Western blotting analysis was used to assess whether the transcriptional regulation of *YAP1* by CDX2 seen in the promoter assays could be translated to the protein level. Caco-2 cells are dependent on CDX2 expression and thus they are not suitable for knockdown experiments [39,44]. However, an intestinal CDX2 knockout cell line was recently constructed using the LS174T colon cancer cell line [45]. We aimed to compare the CDX2 and YAP1 protein expression in intestinal and non-intestinal cells as well as in intestinal cells with or without CDX2, to clarify the regulatory role of CDX2 on the YAP1 protein expression. Glyceraldehyde-3-phosphate dehydrogenase (GAPDH) was used as a control to measure total protein loaded from the confluent Caco-2, HeLa, LS174T, and LS174T CDX2 knockout cells. 

**Figure 7:**
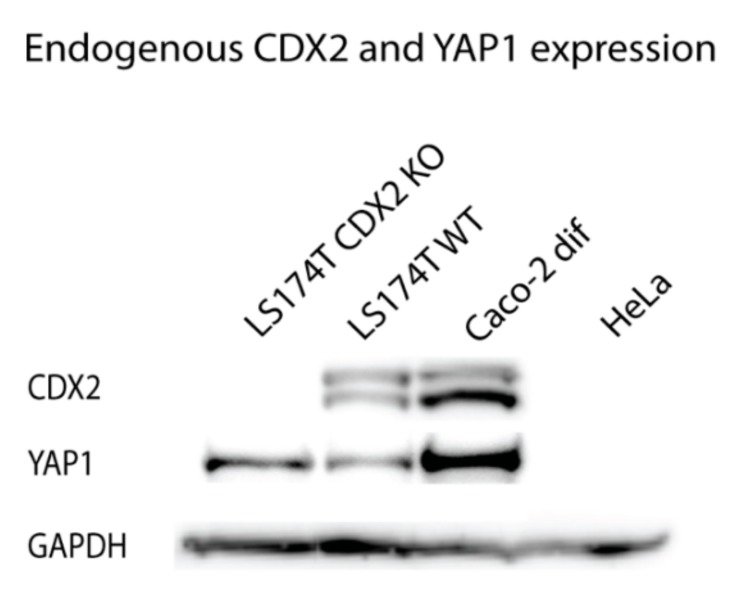
Comparison of CDX2 and YAP1 protein expression using Western blotting, using glyceraldehyde-3-phosphate dehydrogenase (GAPDH) as a reference. Cell lysates are from confluent cells from the intestinal cell lines Caco-2 and LS174T with and without CDX2 knockout (KO), as well as the non-intestinal cell line HeLa. All bands are from the same gel and images were globally adjusted for contrast and brightness.

CDX2 expression was detected in both the LS174T wildtype and Caco-2 cells but not in HeLa cells or the LS174T CDX2 knockout cells (Figure 7). The YAP1 expression was high in Caco-2 cells, lower in LS174T wildtype cells and LS174T CDX2 knockout cells, but was undetected in the non-intestinal HeLa cells. Interestingly, YAP1 expression was present in LS174T knockout cells with no CDX2, demonstrating that CDX2 expression is not essential for YAP1 expression in LS174T cells. This is in line with the finding that the *YAP1* +82 kb enhancer is not active in LS174T cells (Figure 4) and that CDX2, thus, only have limited effect on the basic *YAP1* promoter activity.

## 3. Discussion

The Hippo pathway controls proliferation and differentiation in many tissues partly through YAP1. Dysregulation of YAP1 often leads to development or exacerbation of diseases specific to the tissue in question. While the effects of YAP1 dysregulation in the intestine has been studied, virtually no papers have been published on the transcriptional regulation of *YAP1*. More in-depth knowledge about the regulation of *YAP1* in the intestine will increase the understanding of the role of *YAP1* and the Hippo pathway in the intestine.

Through a combination of bioinformatic analysis of ChIP-seq data and transfection with luciferase promoter-reporter expression constructs containing the *YAP1* promoter, we identified a powerful +82 kb enhancer of *YAP1* that was functional in the intestinal cell lines Caco-2 and T84. However, the +82 kb enhancer did not seem to be functional in four other intestinal cell lines DLD-1, SW480, HT-29, and LS174T (Figure 2). Interestingly, Caco-2 and T84 are the only intestinal cell lines that have the ability to spontaneously differentiate into monolayers of functionally and structurally mature absorptive epithelial cells with microvilli and expression of many brush border enzymes [46,47]. Whereas HT-29 cells are able to differentiate when grown under special culture conditions, LS174T and SW480 cells form multilayers without epithelial polarity or enterocytic differentiation, whatever the culture conditions [47,48,49]. Therefore, we hypothesize that the similarity of Caco-2 and T84 cells to normal intestinal cells might mean that only they have retained the cellular environment needed for the *YAP1* +82 kb enhancer to be functional.

Previous studies have reported SOX2, ERG, TEAD4, and glypican 3 as regulators of *YAP1* expression in osteoprogenitor, hepatic, or prostate tissue. While some detected epigenetic changes to the *YAP1* promoter, none of them identified intragenic regulatory regions or specific binding sites in the *YAP1* gene [7,8,9,10]. Here we present the first clear evidence of an enhancer in the *YAP1* gene that is active in intestinal cells.

We measured the importance of HNF4α and CDX2 for the *YAP1* promoter activity in intestinal cell lines. By mutation of binding sites and promoter/enhancer experiments in Caco-2 cells, we have shown that both CDX2 and HNF4α are involved in activation of the *YAP1* promoter activity. Promoter-reporter assays with mutated CDX2 and HNF4α binding sites in the *YAP1* +82 kb enhancer showed that these sites were important for the enhancer activity (Figure 4). HNF4α overexpression resulted in significantly increased activity of the *YAP1* +82 kb enhancer (Figure 5). It was, however, somewhat surprising that CDX2 overexpression did not increase the reporter gene expression. However, we have previously seen that CDX2 overexpression in Caco-2 cells, which have a high endogenous level of CDX2 [39], does not necessarily result in increased reporter gene expression when CDX2 binds to enhancer regions [43].

HNF4α and CDX2 are essential components of the intestinal transcription factor network that controls development and homeostasis, and CDX2 is normally only expressed in the intestines after birth in mammals [50,51]. During intestinal differentiation, CDX2 and HNF4α are a part of a transcription factor network that tightly regulates a large array of intestinally expressed genes [17,20,36]. *YAP1* is expressed in several tissues but is especially highly expressed in colon cancers [12,13]. We suggest that CDX2 and HNF4α adds an intestinal-specific layer of control to the Hippo pathway through the transcriptional regulation of the *YAP1* gene. 

Using Western blot analysis to measure YAP1 and CDX2 protein expression in LS174T cells with and without CDX2 knockout, we found that CDX2 is not necessary for basic YAP1 expression in these cells. Caco-2 cells have higher levels of both CDX2 and YAP1 protein expression than LS174T. Although the transcriptional regulation of *YAP1* is complex, we suggest that a part of the explanation for increased expression is due to CDX2 binding to the intronic +82 kb enhancer in Caco-2 cells.

By constructing HNF4α knockout intestinal cells lines, it would be possible to determine how important HNF4α is for the general transcription of *YAP1* and its precise contribution to the +82 kb enhancer effect. Although this might not be possible since it has not been possible to find any published work describing an HNF4α knockout intestinal cell line and our lab has not been able to create one either.

In summary, we have identified a promoter region in the *YAP1* gene with importance for the transcriptional activation of *YAP1* in all tested cell lines. Furthermore, we identified a *YAP1* +82 kb enhancer with functional CDX2 and HNF4α binding sites that increases *YAP1* promoter activity in Caco-2 and T84 cells.

## 4. Materials and Methods 

### 4.1. Culture of Human Cell Lines

Caco-2, T84, DLD-1, SW480, HT-29, and LS174T wildtype, colon adenocarcinoma cell line; HEK293, human embryonic kidney cell line; HeLa, cervical adenocarcinoma cell line; LS174T derived CDX2 knockout (LS174T CDX2 knockout) cell line [45] were grown in T175 culture flasks in Dulbecco’s Modified Eagle Medium (Lonza, Basel, Switzerland) or RPMI-1640 for DLD-1 (Thermo Fisher Scientific, Waltham, MA, USA), added 10% Fetal Bovine Serum gold (PAA) and 100 U/mL Penicillin-Streptomycin. Cells were incubated at 37 °C in 5% CO_2_ and passaged every 3–4 days when ~80% confluent. Passaging was done by removing media, rinsing three times with 85 mM sodium citrate, adding 1mL 0.05% trypsin EDTA (Invitrogen, Carlsbad, CA, USA) and incubating 5 min at 37 °C in 5% CO_2_.

### 4.2. Bioinformatic Analysis of the YAP1 Gene

The *YAP1* gene (NM_001130145) was analyzed to identify transcriptionally active regions using the UCSC Genome Browser (Feb. 2009 GRCh37/hg19 assembly). ChIP-seq data were imported as custom tracks from Caco-2 cell precipitates with antibodies for the transcription factors CDX2 and HNF4α as well as for histone acetylation H3K27Ac and methylation H3K4me2 [41]. Additionally, the ENCODE dataset for DNase I activity [42] as well as the UCSC track for 46-way multiple alignment of vertebrate genomes for conservation analysis was also imported. The location of CDX2 and HNF4α binding sites in the *YAP1* promoter region (−0.7 kb to +0.3 kb relative to the TSS; chr11:101,980,493-101,981,464), the intron 1 region (+1.1 kb to +3.2 kb relative to the TSS; chr11:101982249-101983075), the intron 3 region (+67.5 kb to +68.3 kb relative to the TSS; chr11:102048671-102049303) and the intron 4 enhancer (+82.2 kb to +82.8 kb relative to the TSS; chr11:102,063,361-102,063,959) was predicted using an in silico analysis using the online transcription factor database JASPAR (URL: http://jaspar.genereg.net) [52]. The analysis was carried out using the MA0465.1 (URL: http://jaspar.genereg.net/matrix/MA0465.1/) and the MA0114.2 (URL: http://jaspar.genereg.net/matrix/MA0114.2/) matrices for CDX2 and HNF4α, respectively with a relative score cutoff of 75% to 85%. The location of the binding sites in the genome is given relative to the transcription start site (TSS) of *YAP1* at chr11:101,981,192.

### 4.3. Construction of Luciferase Reporter Constructs

The *YAP1* promoter and +82 kb enhancer sequence were PCR amplified and gel purified with primers ordered from Eurofins Genomics (Eurofins Genomics, Ebersberg, Germany) (Table S2-A). pGL4.10 vector (Promega, Madison, WI, USA) was digested with HindIII and gel purified. In-Fusion cloning (Clontech, Fremont, CA, USA) was carried out according to the manufacturer’s protocol, using gel-purified linearized pGL4.10 vectors and promoter insert in a 1:2 molar ratio. 2.5µl cloning reaction was used for transformation of One Shot TOP10 chemically competent E. *coli* (Thermo Fisher Scientific, Waltham, MA, USA). Colonies were isolated and sequenced (Beckman Coulter Genomics, Brea, CA, USA). For the construction of the pGL4.10-*YAP1* promoter+enhancer plasmid, the pGL4.10-*YAP1* promoter plasmid was digested with SalI restriction endonuclease and gel purified, and In-Fusion cloned with enhancer insert using the same procedure.

### 4.4. Construction of Reporter Constructs with Mutated Binding Sites

pGL4.10-*YAP1* promoter+enhancer fragments with mutated binding sites were created by PCR amplifying forward primers containing mutated binding sites, with reverse primers containing In-Fusion tails and vice versa. The products were subsequently combined in a third PCR amplification. Mutated inserts were In-Fusion cloned and transformed. Plasmids were purified and digested with *Pst*I, *Xho*I, or *Xba*I and the fragments were gel purified and sequenced.

### 4.5. Transfection of Cell Lines

Cells were seeded in 24 well plates at 50,000 cells/well (Caco-2, DLD-1, SW-480, and HT-29) or at 100,000 cells/well (HEK293, LS714T, and LS174T CDX2 KO cells) and transfected after 24 h. For each well, a transfection mixture of 50 µl DNA/polyethyleneimine (PEI) mix was prepared, consisting of 2 µM PEI (Alfa Aesar Haverhill, MA, USA) diluted in 150 mM NaCl to 25 µl and 300 ng plasmid DNA diluted with 150 mM NaCl to 25 µl. The transfection mix contained: 25 ng cytomegalovirus (CMV) promoter-driven expression plasmid for CDX2 or HNF4α, 25 ng of an empty CMV expression plasmid that was added to equalize for the amount CMV promoter constructs in the experiments without overexpression, 50 ng promoter-reporter plasmid, 25 ng CMV-LacZ plasmid for internal control of transfection efficiency, and 200 ng pBluescript SK+ II as inactive DNA to reach 300 ng DNA/well. Volume was adjusted with 150 mM NaCl and transfection mix was incubated 60 min at room temperature. The transfection mix was added in a dropwise fashion to wells, followed by light shaking. After transfection, plates were centrifuged at 200 g for 5 min and incubated overnight at 37 °C and 5% CO_2_. The media was changed after 24 h, and cells were grown for an additional 24 h before assayed for luciferase and β-galactosidase activity. All transfection experiments were carried out at least twice with *n* = 4.

### 4.6. Measuring Luciferase Activity of Reporter Constructs

Measurements were carried out on a GloMax® 96 Microplate Luminometer using the Dual-Light™ Luciferase and β-Galactosidase Reporter Gene Assay System (Thermo Fisher Scientific, Waltham, MA, USA). Cells were rinsed three times with 1X phosphate buffered saline (PBS) and lysed with 130 µL TROPIX lysis solution containing 0.5 mM DTT and incubated for 10 min on ice. 10 µL lysate was transferred to a 96 well GloMax Luminometer Light Plate (Promega, Madison, WI, USA), and luciferase activity was measured using a 5-sec integration time and a 2-sec delay. The β-galactosidase activity was measured 45 min after luciferase activity.

### 4.7. Chromatin Immunoprecipitation Assay and Quantitative PCR

Two separate immunoprecipitations from Caco-2 cells were generated and the experiment was run twice with three technical replicates. Both were prepared following the protocol described in [53]. The sets included CDX2, HNF4α, and Hemagglutinin (HA) precipitations and were generated using the antibodies; CDX2 (BioGenex, Freemont, CA, USA, MU392A-UC), HA (Santa Cruz Biotechnology, Santa Cruz, CA, USA, Clone Y-11 X, SC-805 X), or HNF4a (Santa Cruz Biotechnology, Clone SC 171 X, SC-8987 X). qPCR was used to detect binding of CDX2, HNF4α, and HA to the *YAP1* promoter and +82 kb enhancer in the samples, using primers from (Eurofins Genomics, Ebersberg, Germany) (Table S2-B). qPCR was carried out with SsoFast EvaGreen Supermix with Low 6-carboxy-X-rhodamine (ROX) (Bio-Rad, Hercules, CA, USA) on 1 µl (1/20th of total immunoprecipitated (IP) sample DNA) ChIP DNA or H_2_O with 0.5 µM primers on a Stratagene MX3005P real-time thermal cycler (Agilent Technologies, Santa Clara, CA, USA). Relative quantification of ChIP DNA was calculated as a percentage of the input DNA by the delta-delta Ct method [54].

### 4.8. Electrophoretic Mobility Shift Assay

Oligonucleotides for the EMSA was generated with 5′ overhangs and containing either the wildtype binding sequences, a non-specific competitor sequence, or specific competitor sequences with mutated binding sites (Table S3). 250 pmol of each complementary oligonucleotide was annealed in a total volume of 100 µL 0.1 M NaCl by heating to 95 °C followed by passive cooling until room temperature.

For the preparation of radiolabeled oligonucleotides 2.5 pmol annealed oligonucleotide pairs were mixed with 0.5 µL T4 Polynucleotide Kinase, 1 µL 10X forward kinase buffer, 5 µL [γ-32P]-ATP (3000Ci/mmol 5 mCi/ml EasyTide Lead) (PerkinElmer, Waltham, MA, USA) and 2.5 µL H_2_O for a total volume of 10 µL. The reaction was incubated at 37 °C for 30 min after which 20 µL 1X Tris-EDTA (TE) buffer (10 mM Tris-HCl (pH 8.0), 0.1 mM EDTA) was added. The probes were purified with Illustra MicroSpin G-25 Columns (GE Healthcare Life Sciences, Marlborough, MA, USA) and diluted with 1X TE buffer to a total volume of 100 µL and a concentration of about 25 fmol/µL. Radiolabeled oligonucleotides were further diluted 10X in TE buffer immediately before loading.

Caco-2 nuclear extracts used for the EMSA was prepared as previously described [55]. The EMSA reaction contained 1 μl differentiated Caco-2 nuclear extract 4 μl dialysis buffer (25 mM Hepes pH 7.6, 0.1 mM EDTA, 40 nM KCl, and 10% glycerol), 10 μl Gel-shift buffer (25 mM Tris-HCl pH 7.5, 5 mM MgCl_2_, 5% Ficoll 400, 2.5% glycerol, 60 mM KCl, 0.5 mM EDTA, 1 mM DTT, and protease inhibitors), and 0.5 μl dI-dC (homopolymer of deoxyinosine and deoxycytidine). For competition 1 μL 250 fmol/µL unlabeled oligonucleotides, either non-specific, wildtype, or mutated oligonucleotides were added. Supershift assays contained 1 μL antibody; CDX2 (BioGenex, Freemont, CA, USA, MU392A-UC), HA (Santa Cruz Biotechnology, Santa Cruz, CA, USA, Clone Y-11 X, SC-805 X), or HNF4a (Santa Cruz Biotechnology, Santa Cruz, CA, USA, Clone SC 171 X, SC-8987 X). The reaction was incubated 20 min on ice, and then added 1 µL 2.5 fmol of ^32^-P labeled probe followed by 20 min incubation on ice.

Before gel-loading, 2 μl Gel-shift loading buffer (10% glycerol, 0.2% Bromphenol blue and 0.5X Tris-borate-EDTA buffer (2X TBE, 44.5 mM Tris-HCl pH 8.0, 1 mM EDTA, and 44.5 mM boric acid)) was added to the reaction mix. The reaction mix was loaded on a precooled non-denaturing 5% polyacrylamide gel (1 gel: 2.25 mL 30% Acrylamide/Bis-acrylamide (29:1), 0.67 mL 10X TBE, 0.78 mL 87% glycerol, 9.71 mL H_2_O, 54 µL 25% ammonium persulfate (AMPS), and 6.9 µL TEMED), using precooled 0.5X TBE as running buffer. Runtime was 45 min at 100 mV and 25 mA/gel with active cooling. The gel was dried on a slab gel dryer for two hours and exposed on a phosphor-imager for 24 h. The phosphor screen was scanned on a Storm 840 scanner (GE Healthcare Life Sciences, Marlborough, MA, USA) and the image was processed using the Image-Quant Software version 5.2 (GE Healthcare Life Sciences, Marlborough, MA, USA).

### 4.9. Western Blotting

Protein extraction was carried out on cells seeded in 6-well plates at 100,000/well for Caco-2, 300,000/well for LS174T wildtype and LS174T CDX2 KO cells. 48 h after seeding, the media was changed and at 72 h cells were rinsed with cold PBS and lysed for 15 min with 100 µl/well 1X RIPA lysis buffer (1X PBS, 300 mM NaCl, 1% Tergitol NP-40, 0.1% SDS, 0.5% 7-Deoxycholic acid sodium salt, 0.5 µM EDTA pH 8.0) with freshly added 1 mM DTT and 2 µL/mL protease inhibitor mix p8340 (Sigma-Aldrich, St. Louis, MO, USA). Lysates were centrifuged for 20 min at 12.000 g at 4 °C and supernatant saved at −20 °C. Protein concentration was determined by Bradford analysis (Bio-Rad, Hercules, CA, USA).

10 µg or 20 µg protein was mixed 1:4 (v/v) with Bolt loading buffer and 1:10 (v/v) with Bolt sample reducing agent. Samples were incubated at 70 °C for 10 min and loaded on a Bolt 4–12% Bis-Tris Plus gel (Thermo Fisher Scientific, Waltham, MA, USA) along with prestained protein marker PageRuler (Thermo Fisher Scientific, Waltham, MA, USA). SDS-PAGE was performed in 1X Bolt MOPS running buffer, (Thermo Fisher Scientific, Waltham, MA, USA) for 50 min at 150 V. Gels were transferred by wet-electrotransfer to PVDF membranes for 75 min at 25 V and 100 mA in 1X NuPage transfer buffer, (Thermo Fisher Scientific, Waltham, MA, USA). Membranes were blocked with dry skim milk diluted to 5% in Wash buffer (1X PBS with 0.1% Tween-20) for 1 h at room temperature, washed with Wash buffer 3 times for 7 min and incubated overnight at 4 °C with primary antibody diluted in Dilution buffer (2.5% skim milk in Wash buffer). Membranes were washed 3 times for 7 min and incubated with secondary antibody for 1 h at room temperature and washed 3 times for 7 min. Bands were visualized by incubating with the ECL solution SuperSignal™ West Dura Extended Duration Substrate (Thermo Fisher Scientific, Waltham, MA, USA). Antibodies used: CDX2 1:2000, BioGenex, Freemont, CA, USA, MU392A-UC); YAP1 1:15,000, (Abcam, Cambridge, UK, ab52771); GAPDH 1:30,000 (Fitzgerald, North Acton, MA, USA, 10R-G109a), Goat anti-rabbit HRP 1:4000 (Thermo Fisher Scientific, Waltham, MA, USA, 32260); Goat anti-mouse HRP 1:10,000 (Thermo Fisher Scientific, Waltham, MA, USA, 32230).

### 4.10. Statistics

Where applicable, values are represented by mean values with 95% confidence intervals or SD values. The *p*-values have been determined by two-tailed Student’s *t*-tests or one-way ANOVA using GraphPad Prism v7.0 (GraphPad Software, San Diego, CA, USA). The significance levels are shown as * *p* < 0.05, ** *p* < 0.01, *** *p* < 0.001, **** *p* < 0.0001.

## Supplementary Materials

Supplementary Materials can be found at https://www.mdpi.com/1422-0067/20/12/2981/s1.

## Abbreviations

YAP1	Yes-associated protein 1
WWTR1	WW domain containing transcription regulator 1
TEAD	TEA domain transcription factor
ERG	Transcriptional regulator ERG
TEAD4 HA	TEA domain transcription factor 4 Hemagglutinin
PCOS GAPDH	Polycystic ovary syndrome Glyceraldehyde-3-phosphate dehydrogenase
GATA4/5/6	GATA-binding proteins 4/5/6
HNF1A/B	HNF1 homeobox proteins
TCF7L2	Transcription factor 7 like 2
HNF4α	Hepatocyte nuclear factor 4 alpha
CDX2	Caudal type homeobox 2
EMSA	Electrophoretic mobility shift assay
HA	Hemagglutinin
PEI	Polyethyleneimine
AMPS	Ammonium persulfate
