# Supplementary material for: HNF4α and CDX2 Regulate Intestinal YAP1 Promoter Activity
Source: Int J Mol Sci. 2019 Jun 18;20(12):2981. doi: 10.3390/ijms20122981 (PMC6627140; doi:10.3390/ijms20122981)
Supplement: Supplementary file 1 [file ijms-20-02981-s001.zip › S01 supplementary table S1 V06.docx]

**Table S1.** Transcription factor binding sites from an *in-silico* analysis using the online JASPAR tool (URL: <http://jaspar.genereg.net>). *YAP1* input sequence is from the USCS Feb. 2009 GRCh37/hg19 genome assembly. The *YAP1* promoter input sequence spans 972bp at (-0.7kb to +0.3 kb from the TSS; chr11:101,980,493-101,981,464), the *YAP1* enhancer input sequence spans 599bp at (+82.2kb to +82.8kb from the TSS; chr11:102,063,361-102,063,959), the *YAP1* intron 1 input sequence spans 2173bp at (+1.1kb to +3.2kb from the TSS; chr11:101982249-101983075), and the *YAP1* intron 3 input sequence spans 633bp at (+67.5kb to +68.3kb from the TSS; chr11:102048671-102049303). CDX2 and HNF4α sites were predicted in the regions using the MA0465.1 and MA0114.2 matrices and a relative score cutoff of 75% to 85%. The location of the binding sites in the genome is given relative to the transcription start site (TSS) of *YAP1* at chr11:101,981,192.

| **2 HNF4α and 2 CDX2 sites were predicted in the *YAP1* promoter** | | | | | | |
| --- | --- | --- | --- | --- | --- | --- |
| Model ID & name |  | Score | Relative score | Strand | Predicted site sequence | Loc Relative to TSS |
| MA0114.2 HNF4A |  | 7.770 | 0.838 | 1 | TCGGCCTTGGCCCTT | -466 |
| MA0114.2 HNF4A |  | 7.483 | 0.834 | -1 | GAGAACTTTTTCCCT | +111 |
| MA0465.1 CDX2 |  | 6.636 | 0.852 | 1 | GAACAAGAAAA | +34 |
| MA0465.1 CDX2 |  | 7.394 | 0.864 | 1 | AGGAAATAAAG | +44 |
| **2 HNF4α and 3 CDX2 sites were predicted in the *YAP1* enhancer (intron 4 region)** | | | | | | |
| Model ID & name | Site name | Score | Relative score | Strand | Predicted site sequence | Loc Relative to TSS |
| MA0114.2 HNF4A |  | 10.168 | 0.870 | 1 | TTTAACTTTGGTCCT | +82335 |
| MA0114.2 HNF4A | HNF4α | 12.496 | 0.901 | -1 | CTGAACTTTGCTAAC | +82569 |
| MA0465.1 CDX2 | CDX2-S2 | 12.227 | 0.936 | -1 | GTGCAATAAAT | +82554 |
| MA0465.1 CDX2 | CDX2-S1 | 14.121 | 0.965 | -1 | AGGCAATAAAG | +82320 |
| **2 CDX2 and 5 HNF4α sites were predicted in the YAP1 intron 1 region** | | | | | | |
| Model ID & name |  | Score | Relative score | Strand | Predicted site sequence | Loc Relative to TSS |
| MA0465.1 CDX2 |  | 5.829 | 0.840 | -1 | GAGCAAAAAAC | +1335 |
| MA0465.1 CDX2 |  | 3.874 | 0.811 | -1 | GGAAAATAAAT | +1391 |
| MA0114.2 HNF4A |  | 7.915 | 0.840 | +1 | GGGGCCTCTGGCATT | +1157 |
| MA0114.2 HNF4A |  | 6.273 | 0.818 | +1 | TTGAGCCCTGGGCCC | +1213 |
| MA0114.2 HNF4A |  | 6.131 | 0.816 | +1 | TTGGAACTTGGGCCG | +1506 |
| MA0114.2 HNF4A |  | 5.879 | 0.813 | -1 | CAGGACTTGGCCTAT | +1699 |
| MA0114.2 HNF4A |  | 5.329 | 0.805 | -1 | TTGGCCTATGAGTCA | +1705 |
| **2 HNF4α and 4 CDX2 sites were predicted in the YAP1 intron 3 region** | | | | | | |
| Model ID & name | Site name | Score | Relative score | Strand | Predicted site sequence | Loc Relative to TSS |
| MA0114.2 HNF4A |  | 3.813 | 0.785 | +1 | CTGTACTTTGTAGGA | +67841 |
| MA0114.2 HNF4A |  | 1.639 | 0.756 | -1 | ATGACCTTTTAAAGG | +67771 |
| MA0465.1 CDX2 |  | 7.540 | 0.866 | -1 | ATGTCATAACA | +68074 |
| MA0465.1 CDX2 |  | 5.718 | 0.838 | -1 | TGGGAATAAAG | +67900 |
| MA0465.1 CDX2 |  | 5.071 | 0.829 | +1 | GTATAATAATA | +67615 |
| MA0465.1 CDX2 |  | 3.772 | 0.809 | +1 | TCATCATAAAT | +67718 |
