# Supplementary material for: HNF4α and CDX2 Regulate Intestinal YAP1 Promoter Activity
Source: Int J Mol Sci. 2019 Jun 18;20(12):2981. doi: 10.3390/ijms20122981 (PMC6627140; doi:10.3390/ijms20122981)
Supplement: Supplementary file 1 [file ijms-20-02981-s001.zip › S01 supplementary table S1 V06.pdf]

Table S1. Transcription factor binding sites from an *in-silico* analysis using the online JASPAR tool (URL: http://jaspar.genereg.net). *YAP1* input sequence is from the USCS Feb. 2009 GRCh37/hg19 genome assembly. The *YAP1* promoter input sequence spans 972bp at (-0.7kb to +0.3 kb from the TSS; chr11:101,980,493-101,981,464), the *YAP1* enhancer input sequence spans 599bp at (+82.2kb to +82.8kb from the TSS; chr11:102,063,361-102,063,959), the *YAP1* intron 1 input sequence spans 2173bp at (+1.1kb to +3.2kb from the TSS; chr11:101982249-101983075), and the *YAP1* intron 3 input sequence spans 633bp at (+67.5kb to +68.3kb from the TSS; chr11:102048671-102049303). CDX2 and HNF4α sites were predicted in the regions using the MA0465.1 and MA0114.2 matrices and a relative score cutoff of 75% to 85%. The location of the binding sites in the genome is given relative to the transcription start site (TSS) of *YAP1* at chr11:101,981,192.

**2 HNF4α and 2 CDX2 sites were predicted in the *YAP1* promoter**						
Model ID & name		Score	Relative score	Strand	Predicted site sequence	Loc Relative to TSS
MA0114.2 HNF4A		7.770	0.838	1	TCGGCCTTGGCCCTT	-466
MA0114.2 HNF4A		7.483	0.834	-1	GAGAACTTTTTCCCT	+111
MA0465.1 CDX2		6.636	0.852	1	GAACAAGAAAA	+34
MA0465.1 CDX2		7.394	0.864	1	AGGAAATAAAG	+44

**2 HNF4α and 3 CDX2 sites were predicted in the *YAP1* enhancer (intron 4 region)**						
Model ID & name	Site name	Score	Relative score	Strand	Predicted site sequence	Loc Relative to TSS
MA0114.2 HNF4A		10.168	0.870	1	TTTAACTTTGGTCCT	+82335
MA0114.2 HNF4A	HNF4α	12.496	0.901	-1	CTGAACTTTGCTAAC	+82569
MA0465.1 CDX2	CDX2-S2	12.227	0.936	-1	GTGCAATAAAT	+82554
MA0465.1 CDX2	CDX2-S1	14.121	0.965	-1	AGGCAATAAAG	+82320

2 CDX2 and 5 HNF4α sites were predicted in the YAP1 intron 1 region						
Model ID & name		Score	Relative score	Strand	Predicted site sequence	Loc Relative to TSS
MA0465.1 CDX2		5.829	0.840	-1	GAGCAAAAAAC	+1335
MA0465.1 CDX2		3.874	0.811	-1	GGAAAATAAAT	+1391
MA0114.2 HNF4A		7.915	0.840	+1	GGGGCCTCTGGCATT	+1157
MA0114.2 HNF4A		6.273	0.818	+1	TTGAGCCCTGGGCCC	+1213
MA0114.2 HNF4A		6.131	0.816	+1	TTGGAACTTGGGCCG	+1506
MA0114.2 HNF4A		5.879	0.813	-1	CAGGACTTGGCCTAT	+1699
MA0114.2 HNF4A		5.329	0.805	-1	TTGGCCTATGAGTCA	+1705
**2 HNF4α and 4 CDX2 sites were predicted in the YAP1 intron 3 region**						
Model ID & name	Site name	Score	Relative score	Strand	Predicted site sequence	Loc Relative to TSS
MA0114.2 HNF4A		3.813	0.785	+1	CTGTACTTTGTAGGA	+67841
MA0114.2 HNF4A		1.639	0.756	-1	ATGACCTTTTAAAGG	+67771
MA0465.1 CDX2		7.540	0.866	-1	ATGTCATAACA	+68074
MA0465.1 CDX2		5.718	0.838	-1	TGGGAATAAAG	+67900
MA0465.1 CDX2		5.071	0.829	+1	GTATAATAATA	+67615
MA0465.1 CDX2		3.772	0.809	+1	TCATCATAAAT	+67718
