# Supplementary material for: HNF4α and CDX2 Regulate Intestinal YAP1 Promoter Activity
Source: Int J Mol Sci. 2019 Jun 18;20(12):2981. doi: 10.3390/ijms20122981 (PMC6627140; doi:10.3390/ijms20122981)
Supplement: Supplementary file 1 [file ijms-20-02981-s001.zip › S02 supplementary table S2 V02.docx]

**Table S2.** Primers used to produce the luciferase reporter constructs. Primers with In-Fusion overhangs contain the prefix “inf” and the overhang sequences are underlined. Bold text denotes the sequence of restriction sites replacing transcription factor binding sites via site-directed mutagenesis; **B)** Primers used for real-time PCR in the analysis of chromatin immune precipitated DNA from Caco-2 nuclear extract.

| **A)** Primers used for reporter constructs |  |  |
| --- | --- | --- |
| Primer name | Primer Sequence | Restriction site |
| *YAP1*Prom-F | CCGTTTACCCCTCTCAAGTG |  |
| *YAP1*Prom-R | GCTGTCCTCGCTCTCAGG |  |
| Inf-*YAP1*Prom-F | CTCGGCGGCC**AAGCTT**CCGTTTACCCCTCTCAAGTG | HindIII |
| Inf-*YAP1*Prom-R | CCGGATTGCC**AAGCTT**GCTGTCCTCGCTCTCAGG | HindIII |
| *YAP1*enhancer-F | TGACTGGATTAGACTGGATGCT |  |
| *YAP1*enhancer-R | GGAAAAAGAAAATGTAGTGAGAGC |  |
| Inf-*YAP1* enhancer-F | ATAAGGATCC**GTCGAC**TGACTGGATTAGACTGGATGCT | Sal1 |
| Inf-*YAP1*enhancer-R | AAGGGCATCG**GTCGAC**GGAAAAAGAAAATGTAGTGAGAGC | SalI |
|  |  |  |
|  |  |  |
| **B)** Primers used for ChIP analysis |  |  |
| Primer name | Primer Sequence |  |
| YAP1-enhancer F | CCTCTGGTCACAGTGTGGAA |  |
| YAP1-enhancer R | TTGCCTGGACATACTCACCA |  |
