# Supplementary material for: HNF4α and CDX2 Regulate Intestinal YAP1 Promoter Activity
Source: Int J Mol Sci. 2019 Jun 18;20(12):2981. doi: 10.3390/ijms20122981 (PMC6627140; doi:10.3390/ijms20122981)
Supplement: Supplementary file 1 [file ijms-20-02981-s001.zip › S02 supplementary table S2 V02.pdf]

Table S1. Primers used to produce the luciferase reporter constructs. Primers with In-Fusion overhangs contain the prefix "inf" and the overhang sequences are underlined. Bold text denotes the sequence of restriction sites replacing transcription factor binding sites via site-directed mutagenesis; **B)** Primers used for real-time PCR in the analysis of chromatin immune precipitated DNA from Caco-2 nuclear extract.

**A)** Primers used for reporter constructs		
Primer name	Primer Sequence	Restriction site
*YAP1*Prom-F	CCGTTTACCCCTCTCAAGTG	
*YAP1*Prom-R	GCTGTCCTCGCTCTCAGG	
Inf-*YAP1*Prom-F	CTCGGCGGCC**AAGCTT**CCGTTTACCCCTCTCAAGTG	HindIII
Inf-*YAP1*Prom-R	CCGGATTGCC**AAGCTT**GCTGTCCTCGCTCTCAGG	HindIII
*YAP1*enhancer-F	TGACTGGATTAGACTGGATGCT	
*YAP1*enhancer-R	GGAAAAAGAAAATGTAGTGAGAGC	
Inf-*YAP1* enhancer-F	ATAAGGATCC**GTCGAC**TGACTGGATTAGACTGGATGCT	Sal1
Inf-*YAP1*enhancer-R	AAGGGCATCG**GTCGAC**GGAAAAAGAAAATGTAGTGAGAGC	SalI

**B)** Primers used for ChIP analysis	
Primer name	Primer Sequence
YAP1-enhancer F	CCTCTGGTCACAGTGTGGAA
YAP1-enhancer R	TTGCCTGGACATACTCACCA
