# Supplementary material for: HNF4α and CDX2 Regulate Intestinal YAP1 Promoter Activity
Source: Int J Mol Sci. 2019 Jun 18;20(12):2981. doi: 10.3390/ijms20122981 (PMC6627140; doi:10.3390/ijms20122981)
Supplement: Supplementary file 1 [file ijms-20-02981-s001.zip › S03 supplementary table S3 V02.docx]

**Table S3.** Double-stranded oligonucleotides used for electrophoretic mobility shift assay (EMSA) and for constructing pGL4.10-*YAP1* promoter+enhancer constructs with mutated CDX2 and HNF4α binding sites. The wildtype or mutated binding sites are marked in bold.

| Oligos | Sequences 5´-> 3´ |
| --- | --- |
| Non-specific competitor | 5´AACGTAGCTGATCGAATCGGTTAC  3´TGCATCGACTAGCTTAGCCAATGA |
| CDX2-S1 | 5´TTTTGAACTA**TTTATT**GCACCATT  3´AAACTTGAT**AAATAA**CGTGGTAAA |
| CDX2-S1-Mut | 5´AGATACTTTTGAACT**CTGCAG**GCACCATTTAAGTT  3´CTATGAAAACTTGA**GACGTC**CGTGGTAAATTCAAA |
| CDX2-S2 | 5´AAGTTCAGC**TTTATT**GCCTTAAAG  3´TCAAGTCG**AAATAA**CGGAATTTCT |
| CDX2-S2-Mut | 5´TTAGCAAAGTTCAGC**CTCGAG**GCCTTAAAGATGAT  3´ATCGTTTCAAGTCG**GAGCTC**CGGAATTTCTACTAC |
| HNF4α | 5´TTCATGTTAG**CAAAGT**TCAGCTTT  3´AGTACAATC**GTTTCA**AGTCGAAAT |
| HNF4α-Mut | 5´ACATGTTCATGTTAG**TCTAGA**TCAGCTTTATTGCC  3´GTACAAGTACAATC**AGATCT**AGTCGAAATAACGGA |
