# Supplementary material for: HNF4α and CDX2 Regulate Intestinal YAP1 Promoter Activity
Source: Int J Mol Sci. 2019 Jun 18;20(12):2981. doi: 10.3390/ijms20122981 (PMC6627140; doi:10.3390/ijms20122981)
Supplement: Supplementary file 1 [file ijms-20-02981-s001.zip › S03 supplementary table S3 V02.pdf]

Table S3. Double-stranded oligonucleotides used for electrophoretic mobility shift assay (EMSA) and for constructing pGL4.10-*YAP1* promoter+enhancer constructs with mutated CDX2 and HNF4α binding sites. The wildtype or mutated binding sites are marked in bold.

Oligos	Sequences 5´-> 3´
Non-specific competitor	5´AACGTAGCTGATCGAATCGGTTAC<br>3´TGCATCGACTAGCTTAGCCAATGA
CDX2-S1	5´TTTTGAACTA**TTTATT**GCACCATT<br>3´AAACTTGAT**AAATAA**CGTGGTAAA
CDX2-S1-Mut	5´AGATACTTTTGAACT**CTGCAG**GCACCATTTAAGTT<br>3´CTATGAAAACTTGA**GACGTC**CGTGGTAAATTCAAA
CDX2-S2	5´AAGTTCAGC**TTTATT**GCCTTAAAG<br>3´TCAAGTCG**AAATAA**CGGAATTTCT
CDX2-S2-Mut	5´TTAGCAAAGTTCAGC**CTCGAG**GCCTTAAAGATGAT<br>3´ATCGTTTCAAGTCG**GAGCTC**CGGAATTTCTACTAC
HNF4α	5´TTCATGTTAG**CAAAGT**TCAGCTTT<br>3´AGTACAATC**GTTTCA**AGTCGAAAT
HNF4α-Mut	5´ACATGTTCATGTTAG**TCTAGA**TCAGCTTTATTGCC<br>3´GTACAAGTACAATC**AGATCT**AGTCGAAATAACGGA
